# Hybrid purity assessment in *Eucalyptus* F_1_ hybrids using microsatellite markers

**DOI:** 10.1007/s13205-013-0161-1

**Published:** 2013-08-27

**Authors:** V. Subashini, A. Shanmugapriya, R. Yasodha

**Affiliations:** Division of Plant Biotechnology, Institute of Forest Genetics and Tree Breeding, Coimbatore, 641002 India

**Keywords:** *Eucalyptus tereticornis*, *Eucalyptus camaldulensis*, Inter-specific hybrids, Simple sequence repeats, Genotyping, Parentage confirmation

## Abstract

The worldwide expansion of hybrid breeding and clonal forestry is to meet the demands of paper pulp and bioenergy. Although India was one of the pioneers in hybrid production of eucalypts only recently the hybrid clonal forestry is gaining momentum. Inter-specific hybrids are being produced to exploit the hybrid vigor of F_1_ individuals. Quality control genotyping for hybrid purity and parentage confirmation at the early stage is one of the essential criteria for clonal propagation and field trails for the assessment of growth performance. Eucalyptus being a obligatory outcrossed species with potential to self pollination, possibilities of pollen contamination are high. Hence, in the present study, *Eucalyptus camaldulensis* × *E. tereticornis* inter-specific hybrids were genotyped using 25 fluorescent labeled microsatellite markers available in public domain. Multiplex loading of PCR products was performed successfully for most of the microsatellite loci. Hybrid purity index was calculated and parentage was confirmed. Hybrid purity values ranged from 85 to 100 % showed the efficiency of controlled pollination techniques. A subset of six fully informative simple sequence repeats was identified for routine quality control genotyping for these hybrids. Detection of non-essential genotypes observed among the hybrid seedlings proved the significance of hybrid purity tests and the false hybrids were removed at the seedling stage. The hybrids with proven hybridity will be used for generation of genetic linkage, discovery of quantitative trait loci and the individuals with high productivity can enter into mass clonal multiplication.

## Introduction

Hybrid forestry in eucalypts is a success story and currently several eucalypt growing countries are adopting hybrid breeding for industrial plantations. The greatest advance in industrial plantation forestry has undoubtedly been in the clonal deployment of F_1_ hybrid genotypes. Clonal propagation and hybrid breeding have become a powerful combination of tools for the improvement of production and wood quality in eucalypts (Grattapaglia and Kirst 2008). Reports on controlled inter-specific hybrid eucalypts production and their importance in genetic improvement were available since 1960 in India for the commercially important species like *Eucalyptus camaldulensis*, *Eucalyptus tereticornis* and *Eucalyptus grandis* (Venkatesh and Kedharnath 1965; Venkatesh and Sharma 1977, 1979). However, eucalypts breeding program in India mainly focused on improvement of pure species (Varghese et al. 2009) which is obviously a prerequisite for hybrid development. Many earlier hybrid selections in Congo and Brazil were from sporadic hybrids identified from open pollinated seed orchards and later shifted to planned attempts for production of inter-specific hybrids (Potts and Dungey 2004). The Indian land race of *E. tereticornis* (Mysore gum) was evolved from natural hybrid and suffers from hybrid breakdown or inbreeding depression, necessitating the production of targeted crosses for hybrid vigor. A combination of complementary traits from *E. grandis* and *E. urophylla* has great potential for expanding genetic diversity, for improving pulp properties and for introducing adventitious rooting traits in F_1_ hybrids. Subsequently, in India attempts are being made to generate inter-specific crosses among *E. camaldulensis*, *E. tereticornis* and *E. grandis* with phenotypically characterized parents (http://ifgtb.icfre.gov.in/news&events/Eucalypts%20Hybrid%20Breeding%20programme.pdf). Such hybrids are essential to map genes controlling various economically important traits for early screening of hybrid progenies with the right combination of desirable characteristics and many other molecular genetic studies. Further, many countries working eucalypt breeding programs also contemplate on marker assisted selection (MAS) in eucalypts, which would aid indirect selection of difficult traits at early stage without waiting for next generation. Thus, MAS speeds up the process of conventional breeding and facilitates improvement of traits which is difficult through conventional breeding. Hybridity validation at the seedling stage is one of the essential criteria for inter-specific hybrid production and establishment of true segregating population. The hybrid authentication procedures identify the pollen contamination, out crossing with foreign pollens and physical admixtures during seed handling at storage and nursery raising stages. Thus use of individuals with erroneous hybridity results in segregation of the traits, lower yields and genetic deterioration of clones. Further, exploitation of heterosis in eucalypts requires sustained production of F_1_ hybrids and management of hybrid identity during clonal propagation.

Quality control (QC) genotyping is an important aspect in eucalypt improvement program, however, no well-established methods available for implementation in hybrid breeding program. The use of DNA markers particularly Simple Sequence Repeats (SSRs) are useful for a variety of molecular breeding applications because of their codominance, abundance, high genome coverage and multi allelic nature. SSRs are the most suitable markers for hybrid purity assessment as the heterozygosity of the hybrids can be easily determined by the presence of alleles from both the parents used for controlled pollination. They are already a proven tool for hybrid authentication or hybrid purity assessment and parentage confirmation in many crop species (Bohra et al. 2011). Indeed, molecular markers-based hybrid purity tests have been developed and are in routine use in many crop species such as rice (Yashitola et al. 2002; Sundaram et al. 2008), maize (Asif et al. 2006), cotton (Selvakumar et al. 2010) and safflower (Naresh et al. 2009). Similar to food crops, in forest trees also inter-specific hybrids are generated routinely through hybridization and the heterotic individuals are selected for clonal propagation and mass multiplication (Nikles and Griffin 1992; Stanton et al. 2010). SSRs were recommended for species and hybrid identification, certification of controlled crosses in species such as *Populus* (Rajora and Rahman 2001), Tsuga (Bentz et al. 2002), Picea (Narendrula and Nkongolo 2012) and *Pinus* (Elliott et al. 2006). Recently, in eucalypts, reasonably good numbers of SSR markers become available (Brondani et al. 2006; Acuna et al. 2012; He et al. 2012; Faria et al. 2010) and these SSRs can be used for developing molecular marker based hybrid purity test. Hence, the objectives of this study were to identify SSR markers for confirmation of parentage and hybrid purity tests in *E. tereticornis* × *E. camaldulensis* and identify a subset of highly informative SSR markers for routine and low-cost quality control genotyping.

## Materials and methods

### Plant material

Juvenile leaves were collected from the parent trees of *E. camaldulensis* (Clone 7) and *E. tereticornis* (Clone 88) developed through the breeding program of Institute of Forest Genetics and Tree Breeding (IFGTB), Coimbatore. In the case of F_1_ inter-specific hybrids (*E. camaldulensis* × *E. tereticornis*), the juvenile leaves were collected from 6-month-old plants. Genomic DNA was extracted from 100 mg of the plant sample using Qiagen DNeasy Plant Mini Kit^TM^. The extracted DNA quality of each sample was assessed using 0.8 % agarose gel and the quantity of DNA was confirmed using Picodrop spectrophotometer (Picodrop microlitre spectrophotometer version 3.01, UK) and DNA concentrations were normalized at 10 ng/μl.

### Polymerase chain reaction (PCR)

SSR locus specific forward primer was designed with a 5′-end M13 tail (5′ TGT AAA ACG ACG GCC AGT-3′) as reported by Schuelke (2000). Initially, to identify the polymorphic SSRs between the parents, 53 SSR markers located on the 11 linkage groups selected from the SSR set developed and mapped by Brondani et al. (2006) from *E. grandis* and one loci from *E. grandis* mapped by Thamarus et al. (2004) were considered. PCR amplification was carried out in 10 μl volume with 10 × buffer containing 100 mM Tris–HCl (pH 8.3), 500 mM KCl and 15 mM MgCl_2_, 0.4 pmol of each forward (M13 tailed) and reverse primer, 1 U of *Taq* DNA polymerase, and 100 μM of each dNTPs and 20 ng of template DNA. The PCR amplification was carried out for 5 min at 94 °C, 30 cycles of 1 min at 94 °C, 60 or 30 s at the primer specific annealing temperature, 2 min at 72 °C, and 15 min at 72 °C for final extension. Annealing temperatures varied from 48 to 60 °C, to amplify specific microsatellite marker. PCR products were size-separated using an 5 % denaturing polyacrylamide (PAGE) gels of size 21 × 50 cm (Sequi-Gen GT System, BIO-RAD, USA) containing 7 M urea and 1 × TBE buffer, and visualized by silver staining. SSR loci showing polymorphism between parents were used for genotyping of the F_1_ hybrid population. PCR amplification was carried out in 10 μl volume with 10 × buffer containing 100 mM Tris–HCl (pH 8.3), 500 mM KCl and 15 mM MgCl_2_, 125 μM dNTP mix, 0.1 pmol of M13 tailed forward primer, 0.4 pmol of reverse primer and 0.2 pmol of fluorescently labeled [6-FAM^TM^, NED^®^, VIC^®^,or PET^®^ (Applied Biosystems, CA, USA)], 10 ng of template DNA and 1 U of *Taq* DNA polymerase. The touchdown PCR program used was as follows: initial denaturation at 94 °C for 5 min, then 30 cycles of 94 °C for 45 s, annealing temperature varying from 48 to 60 °C for 30 s and 72 °C for 1 min and an elongation step at 72 °C for 15 min. After these cycles, an additional round of 20 cycles with 94 °C for 30 s, annealing of labeled M13 at 50 °C for 45 s and 72 °C for 45 s, and final extension of 72 °C for 30 min.

PCR products were diluted specifically for each dye wherein an aliquot of 1 μl of fluorescently labeled PCR product was mixed with 0.2 μl of GeneScan^TM^ 600-LIZ-Size^®^ Standard V2.0. (Applied Biosystems) and 8.8 μl of Hi-Di formamide (Applied Biosystems). The mixture was electroinjected in ABI 3500 genetic analyzer (Applied Biosystems). Data obtained after 45 min of injection were analyzed with Genemapper^TM^ software version 4.1 (Applied Biosystems) and exported as data table.

Multiplexing in the form of multiloading was carried out by mixing two or three SSRs amplified with different fluorescent dye or two SSRs having different fragment sizes amplified with same dye (Tables 1, 2). While loading more than one PCR product, the concentration of the each product was adjusted (0.5 or 1.0 μl) to avoid off-scale peaks. Only two loci were loaded singly.

**Table 1:** Details of SSR loci used for genotyping of eucalypt hybrids

S. no.	Microsatellite	Repeat motif	Annealing temperature (in °C)	Size (bp)	Fluorochrome^A^
1	Embra12	(AG)_22_	59	130–150	VIC
2	Embra36	(AG)_29_	55	150–170	FAM^a^
3	Embra98	(AG)8(G)6(AG)3AA… (GA)20	56	240–270	FAM^b^
4	Embra122	(GA)_26_	56	230–280	FAM^a^
5	Embra147	(AG)*n*	56	184–210	FAM
6	Embra89	(CT)17	54	300–310	FAM^c^
7	Embra33	(AG)19	58	125–160	FAM^b^
8	Embra101	(AG)_12_A(AG)_8_	55	130–155	FAM^c^
9	Embra227	(GA)_13_	62	305–340	FAM^d^
10	Embra206	(GA)8AA(GA)11	57	305–335	FAM
11	Embra6	(AG)_19_	58	140–155	FAM^d^
12	Embra186	(GA)27	58	160–190	NED
13	Embra20	(AG)_19_	59	145–170	PET
14	Embra58	(AG)_20_	59	155–180	VIC
15	Embra213	(CT)17	53	220–240	PET
16	Embra28	(AG)_25_	56	190–210	FAM
17	Embra97	(CT)21	58	130–150	NED
18	Eg16	(GA)_12_(TGA)_2_	60	245–260	FAM
19	Embra179	(GA)_9_	56	145–160	VIC
20	Embra303	(CT)20	56	270–300	NED
21	Embra53	(AG)17GT(AG)5	60	130–150	NED
22	Embra304	(GA)35(GGA)4	54	225–245	VIC
23	Embra52	(AG)2T(AG)26	56	120–160	VIC
24	Embra18	(AG)_3_GG(AG_)19_	52	130–140	NED
25	Embra167	(TC)_19_	48	120–140	FAM

^A^Fluorochrome with same letters was multiplexed together for electrophoresis

**Table 2:** Combination SSR loci used for multiplex loading of SSR-PCR

S. no.	Fluorochrome			
	FAM	NED	VIC	PET
1	Eg16	Embra97	Embra304	–
2	–	Embra53	Embra179	–
3	–	Embra18	Embra52	–
4	Embra167	Embra303	–	–
5	Embra28	Embra120	–	Embra20
6	–	Embra186	Embra12	Embra213

### Statistical analysis

The allele size data obtained from the Genemapper for all loci were analyzed using Powermarker software to estimate Polymorphic Information Content (PIC). Hybrid purity assessment was carried out wherein the SSR allele data for the hybrid seeds were recorded as “A” [allele of male parent (*E. tereticornis*)], “B” [allele of female parent (*E. camaldulensis*)] and “H” (alleles from both the parents “Hybrid”) format. Purity index for each marker was calculated using scored data by applying the following formula (Bohra et al. 2011):

$$\text{Purity}\;\text{index}\;\left(\%\right)=\frac{\text{Number}\;\text{of}\;\text{true}\;\text{hybrids}\left(\text{containing}\;\text{alleles}\;\text{of}\;\text{both}\;\text{the}\;\text{parents}\right)}{\text{Total}\;\text{number}\;\text{of}\;\text{hybrid}\;\text{seeds}\;\text{tested}}\times 100$$

## Results and discussion

In agricultural crops, hybrid genetic purity is a routine exercise and DNA markers are often used for quality control genotyping (Wu et al. 2006; Selvakumar et al. 2010; Mbanjo et al. 2012; Semagn et al. 2012). In forest tree species, intentional hybrid breeding is restricted to few species like eucalypts (Potts and Dungey 2004; Dickinson et al. 2013), *Acacia* (Griffin et al. 2010), *Populus* (Yu et al. 2001) and *Pinus* (Cappa et al. 2013). Hybrid seed production in eucalypts is generally takes the advantage of the natural protandry and controlled pollination (CP) is usually carried out by hand emasculation and pollination. During this process, the genetic purity of the F_1_ seeds is significantly affected by foreign pollen or self pollen. The high cost of performing CP activity and relatively low seed production coupled with large scale clonal propagation of specific hybrids demands the quality control for parentage. Unlike agricultural crops, in tree species, Grow Out Tests (GOTs) for morphological assessment cannot be practiced due to the long duration. The DNA markers such as SSRs could be of excellent choice for hybridity confirmation in eucalypts, which has good resources of SSRs. Hybrid purity assessment would also support the stringent intellectual property requirements for variety registration under plant protection of varieties and farmer’s right authority of India (PPV & FRA); and the SSR marker technologies have already been in use for accurate identification of eucalypts in Brazil wherein for eucalypts clonal protection requests are accompanied by a multilocus DNA profile (DNA fingerprint) of 15–20 microsatellite markers that were recommended based on several aspects such as robustness, polymorphic information content and general availability in the public domain (Grattapaglia 2007) and South Africa (http://www.forestry.co.za/application-of-plant-breeders-rights/).

Preliminary results showed that among the 53 SSR loci analyzed, 25 loci were suitable for further studies and remaining 28 loci showed either large number of non-essential genotypes or no amplification products with more than 20 % of F_1_ individuals. These 28 loci required further standardization in fluorescent labeled PCR and hence discarded for further analysis. The details of SSR loci and the information on PCR amplification status, genotype pattern, hybrid purity index and the polymorphic information content for each locus is given in Table 3. Among the selected 25 loci, the SSR primer set Eg16 could not amplify 16 F_1_ individuals and EMBRA303 produced maximum number of (27) non-essential genotypes, i.e., non-specific PCR products. The PIC values were ranging from 0.4 to 0.8.

**Table 3:** Hybrid purity index and polymorphic information content of different SSR loci used for genotyping *Eucalyptus* mapping population

Marker name	Genotype of parents	Number of individuals						Purity of designated hybrid (%)	PIC
		Lacking amplified product	Amplified product	Having allele from parent A	Having allele from parent B	Identified as hybrid	Non-essential genotypes		
Embra12	abxac	4	100	0	0	100	0	100.00	0.5
**Embra36**	abxcd	4	100	0	0	100	0	100.00	0.7
**Embra98**	abxcd	5	99	0	0	98	1	98.99	0.6
Embra122	abxcc	1	103	0	2	101	0	98.06	0.5
Embra147	abxcc	2	102	2	0	100	0	98.04	0.6
Embra89	abxaa	5	99	0	0	97	2	97.98	0.4
Embra33	abxbc	6	98	0	0	96	2	97.96	0.5
Embra101	abxac	2	102	0	0	101	1	99.02	0.5
**Embra227**	abxcd	8	96	0	2	94	0	97.92	0.7
Embra206	abxbc	6	98	0	0	98	0	100.00	0.5
**Embra6**	abxcd	5	99	3	0	95	1	95.96	0.7
Embra186	abxcd	8	96	2	0	94	0	97.92	0.5
Embra20	abxcd	2	102	0	2	100	0	98.04	0.7
Embra58	abxcd	13	91	0	2	89	0	97.80	0.7
**Embra213**	abxcd	3	101	1	0	100	0	99.01	0.7
Embra28	abxcd	3	101	6	0	90	5	89.11	0.8
Embra97	aaxbb	14	90	2	2	85	1	94.44	0.4
Eg16	abxac	16	88	0	0	88	0	100.00	0.6
Embra179	abxbc	13	91	0	0	91	0	100.00	0.6
Embra303	abxcd	8	96	2	0	90	4	93.75	0.8
Embra53	abxcd	8	96	0	1	93	2	96.88	0.7
Embra304	abxac	8	96	0	0	95	1	98.96	0.5
Embra52	abxcd	14	90	0	2	78	10	86.67	0.8
Embra18	abxbc	12	92	1	0	85	6	92.39	0.6
**Embra167**	abxcd	8	96	1	0	95	0	98.96	0.7

* Loci marked in bold form a subset for hybrid confirmation and quality control genotyping in clonal propagation

**Fig. 1:**
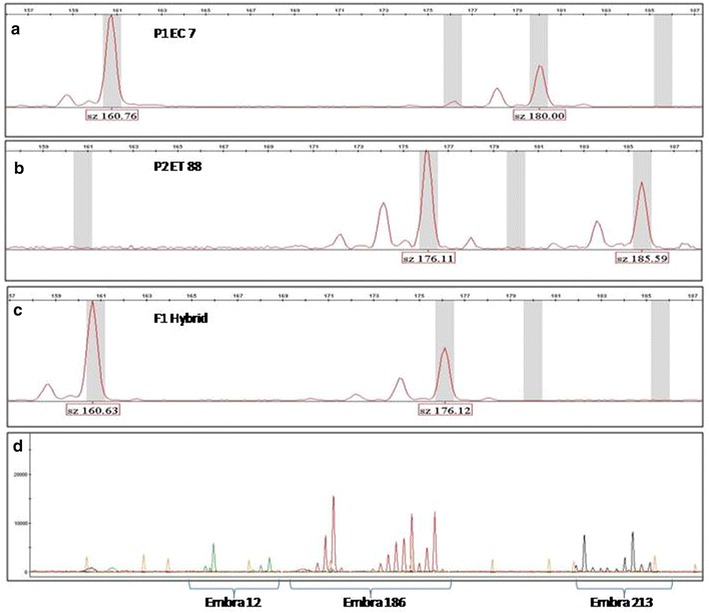
Electropherogram of SSRs obtained with software Genemapper. Female parent *E. camaldulensis* (**a**), Male parent *E. tereticornis* (**b**) and F1 Hybrid amplified with locus EMBRA186 (**c**). Multi-loading of three loci amplified with different fluorescent dye EMBRA12 (VIC), EMBRA186 (PET) and EMBRA213 (NED) in a single capillary (**d**)

Steenkamp et al. (2003) made an attempt to detect putative hybrids in commercially propagated eucalyptus using 5S rDNA sequence, however, it appears to be unsuitable for constructing species–specific PCR-primers because of the variable sites in the 5S repeat. Similarly, Cupertino et al. (2009) used six SSRs for parentage analysis in multiple hybrid families of eucalypts and found pollen contamination and mislabeling thus warranting parentage confirmation for all controlled crosses. In the present study, hybrid purity assessment was carried out for the F_1_ hybrid progenies obtained from *E. camaldulensis* × *E. tereticornis* using the EMBRA SSR markers. The fragment analysis was performed as a multi-loading assay, analyzing two or three loci simultaneously that was labeled by different fluorescence dyes or with loci of different fragment sizes amplified with single fluorescence dye (Fig. 1). This could increase the amount of information generated per assay, and reduced consumable costs. Cost effective genotyping by fluorescence allowed more rapid data collection in many plant breeding experiments compared to earlier methods such as those based on radioactive isotopes and silver staining (Guichoux et al. 2011). Hybrid purity values for all 25 loci were >85.0 % and few loci showed up to 10 non-essential genotypes.

### Selection of a set of SSRs for hybrid quality control in clonal propagation

To identify a set of informative markers that could be used for routine genotyping of hybrids for QC, minimum number of SSR loci was determined based on the parentage confirmation and amplification pattern. Subset of six fully informative SSRs with four different alleles (Table 3) would be sufficient to verify the hybridity and for quality control during clonal multiplication of these hybrids. Use of fluorescent-based SSR genotyping in hybrid purity assessment generates specific and accurate data for hybrid identification and subsequently when these hybrids enter clonal propagation chain these SSR markers could be useful in confirming the identity.

## Conclusion

Generally, F_1_ hybrids contain DNA from both the parents and SSR markers identified both male and female parent–specific markers allowing differentiation of true eucalypts hybrids from selfed individuals and outcrossed individuals with foreign pollen. Presence of non-essential genotypes observed among the hybrid seedlings established the importance of hybrid purity tests and those false hybrids could be removed at the early stage of multiplication. The SSR marker information developed through this study will be of immense help for hybrid eucalypts industry to select appropriate marker combinations and assess hybrid purity of the clones. Other possible applications of hybrid purity assessment include development of genetic linkage maps, quantitative analysis of economically important traits, and marker assisted selection where true and pure hybrids are essential.
